# Risk factors for manifestations of frailty in hospitalized older adults: A qualitative study

**DOI:** 10.1111/jan.15120

**Published:** 2021-11-30

**Authors:** Faye Wray, Susanne Coleman, David Clarke, Kristian Hudson, Anne Forster, Elizabeth Teale

**Affiliations:** ^1^ University of Leeds Leeds UK; ^2^ Bradford Teaching Hospitals NHS Foundation Trust Bradford UK

**Keywords:** acute care, focus groups, frailty, interviews, nurses, nursing, qualitative, risk factor

## Abstract

**Aims:**

To explore the experiences of older people and ward staff to identify modifiable factors (risk factors) which have the potential to reduce development or exacerbation of manifestations of frailty during hospitalization. To develop a theoretical framework of modifiable risk factors.

**Design:**

Qualitative descriptive study.

**Methods:**

Qualitative interviews with recently discharged older people (*n* = 18) and focus groups with ward staff (*n* = 22) were undertaken between July and October 2019. Data were analysed using directed content analysis.

**Results:**

Themes identified related to attitude to risk, communication and, loss of routine, stimulation and confidence. Using findings from this study and previously identified literature, we developed a theoretical framework including 67 modifiable risk factors. Risk factors are grouped by patient risk factor domains (pain, medication, nutritional/fluid intake, mobility, elimination, infection, additional patient risk factors) and linked care management sub‐domains (including risk factors relating to the ward environment, process of care, ward culture or broader organizational set up). Many of the additional 36 risk factors identified by this study were related to care management sub‐domains.

**Conclusion:**

A co‐ordinated approach is needed to address modifiable risk factors which lead to the development or exacerbation of manifestations of frailty in hospitalized older people. Risk assessment and management practices should not be duplicative and, should recognize and address modifiable risk factors which occur at the ward and organizational level.

**Impact:**

Some older people leave hospital more dependent than when they come in and this is, in part, due to the environment and process of care and not just the severity of their presenting illness. Many of the risk factors identified need to be addressed at an organizational rather than individual level. Findings will inform a programme of research to develop and test a novel system of care aimed at preventing loss of independence in hospitalized older people.

## 1 INTRODUCTION

Older people remain major users of hospital care; with people over 65 accounting for over 2 million unplanned hospital admissions and 40% of hospital bed days in England each year (Imison et al., 2012; Soong et al., 2015; The Health Foundation, 2018). Whilst some older people recover well from acute illness, others experience physical and functional decline, even when the illness which caused hospitalization is successfully treated (Covinsky et al., 2011; Lafont et al., 2011).

At particular risk are older people living with frailty; an abnormal health state characterized by poor physiological reserve (Clegg et al., 2013). In the 1960s, Bernard Isaacs described five ‘Geriatric Giants’; key syndromes that commonly occur during acute illness in frail older people: falls, delirium, incontinence, immobility, loss of function (Isaacs, 1992). During hospitalization, older people are at increased risk of development or exacerbation of these five ‘manifestations of frailty’ (MoF). MoF are associated with poor outcomes in the short‐term (e.g. in hospital morbidity, hospital‐acquired infection, injurious falls and pressure ulcers) and in the longer term (e.g. increased likelihood of hospital readmission, reduced quality of life and increased levels of dependence; Bagshaw et al., 2014; Cunha et al., 2019; Hubbard et al., 2017; Keeble et al., 2019; Shin et al., 2016). Frail patients are at higher risk of poor outcome compared with non‐frail patients, irrespective of illness severity (Pulok et al., 2020; Romero‐Ortuno et al., 2016).

### 1.1 Background

Decompensated frailty occurring during hospitalization may in part be due to the physiological stresses of acute illness (Clegg et al., 2013). However, there may also be modifiable factors encountered during periods of hospitalization which may contribute to the five MoF. Such factors can often be iatrogenic (i.e. related to the process or organization of hospital care). For example, even if able to ambulate, older people spend much of their time lying in bed or sitting during hospitalization, putting them at risk of immobility and functional decline (Brown et al., 2004; Pedersen et al., 2012). Modifiable factors also have significant overlap and interdependency in terms of their relationship to the development or exacerbation of MoF. For example, immobility (and it's risk factors) puts older people at higher risk of developing delirium (Ahmed et al., 2014); prescription of certain psychoactive medications or drugs with sedative properties can contribute to increased risk of both delirium and falls (Ahmed et al., 2014; Oliver et al., 2004). The relationship between risk factors and tendency to develop MoF is therefore complex, and specific to the individual.

The multidisciplinary care team (and in particular, nurses), has a key role in undertaking risk assessment and management procedures in hospitalized older adults (Han et al., 2021; Redley & Raggatt, 2017). In the United Kingdom, there are separate guidelines for the prevention and management of MoF in hospital e.g. delirium, falls, incontinence (National institute for Health & Care Excellence, 2019a, 2019b, 2019c). These guidelines may result in each MoF being considered in isolation from the others, despite overlapping risk factor profiles for each MoF. This can lead to duplicated assessment and overlapping care pathways. Furthermore, evidence from national audits suggests that risk assessment and management practices may be poorly implemented in practice (Royal College of Physicians, 2012, 2021; Royal College of Psychiatrists, 2017). A co‐ordinated approach to reduce the risk of functional decline in hospitalized older adults is needed. Such an approach should also co‐ordinate with other procedures such as the Comprehensive Geriatric Assessment to ensure that care to reduce the risk of in hospital decline is considered as part of a broader, long‐term, medical, social and functional needs assessment (Parker et al., 2018).

To address this, we undertook a programme of work as part of a National Institute for Health Research (NIHR) Project Development Grant (PDG, reference: RP‐DG‐0218‐10001), ‘Older People: a study to investigate maintaining Independence through a novel system of care (OPTIMISE), aimed at reducing the development or exacerbation of MoF’. To contribute towards the development of the intervention, we sought to identify and prioritize modifiable risk factors for the five MoF to be targeted by the system of care.

Initially we identified modifiable and non‐modifiable risk factors for the five MoF through a scoping review of the literature informed by key guidelines. A summary of the risk factors identified are shown in Table 1. Whilst the scoping review provided a good starting point, we recognized that older people, their family members and ward staff may be in a unique position to identify features of ward environments, practices and organizational structures, not previously identified in the literature which might act as risk factors for MoF. We sought to explore these experiences as part of the current study and to develop a theoretical framework of risk factors.

**TABLE 1:** Risk factors identified by scoping review of literature

	Risk factor	Delirium	Falls	Incontinence	Immobility	Loss of function
Non modifiable	Age	✓	✓	✓	✓	✓
	Cognitive impairment/delirium	✓	✓	✓		✓
	Illness severity	✓			✓	✓
	Co‐morbidity	✓	✓	✓ (stroke)	✓	✓
	Fracture at presentation	✓			✓	
	Previous fall		✓		✓	
Modifiable Patient‐related factors	Visual impairment/not wearing glasses	✓	✓			✓
	Hearing impairment	Evidence gap				✓
	Polypharmacy	✓	✓			✓
	Benzodiazepines	✓	✓	✓	✓	✓
	Anticholinergic drugs	✓	✓			
	Opiates	✓			✓	✓
	Antihypertensives	(dihydropyridines) ✓	✓	✓ (diuretics)		
	Diuretics		✓	✓		
	Psychotropic drugs		✓		✓	
	High fluid intake			✓		
	Dehydration	✓				
	Electrolyte disturbance	✓				
	Depression	Evidence gap			✓	✓
	Infection	✓		✓ (UTI)	✓	
	Incontinence	Evidence gap	✓			
	Bladder catheter	✓	✓	✓	✓	✓
	Urinary retention	✓		✓		
	Pain	✓		✓	✓	✓
	Low BMI/poor nutritional intake				✓	✓
	Footwear		✓			
	Mobility problems		✓	✓	✓	✓
	Balance problems		✓		✓	✓
	Syncope		✓			
Modifiable – due to ward culture						
	Caffeinated drinks			✓		
	Physical or chemical restraints	✓		✓	✓	✓
	Drips, lines, monitors	✓	✓	✓	✓	✓
	Sleep disturbance	✓			✓	
	Prolonged bed rest			✓	✓	✓
	Lack of correct walking aid		✓	✓	✓	✓
Modifiable – time/resource dependent	Delays to answering call bells		✓	✓	✓	
	Room changes	✓				
Modifiable – environmental factors	Isolation				✓	✓
	No clock or watch	✓				
	Incorrect equipment (chairs, walkers)		✓		✓	✓
Hard to modify – environment (estates)	Flooring		✓		✓	
	Lighting	✓	✓			
	Furniture and fittings		✓	✓	✓	✓
	Unfamiliar environment	✓	✓	✓	✓	✓

## 2 THE STUDY

### 2.1 Aims

To explore the experiences of older people and ward staff to identify modifiable factors (risk factors) which have the potential to reduce development or exacerbation of MoF during hospitalization (and physical and functional decline post‐discharge). Using these experiences and informed by the literature, to then develop a theoretical framework of modifiable risk factors.

### 2.2 Design

A qualitative descriptive design was chosen for this study which involved interviews with older people who had recently been discharged from hospital (study one) and focus groups with ward staff (study two; Kim et al., 2017; Lambert & Lambert, 2012; Sandelowski, 2000). We chose this design in line with our research aims which were exploratory and required straightforward descriptions of risk factors which stayed close to the experiences and perceptions of those who participated in the study. The study is reported with reference to the consolidated criteria for reporting qualitative studies (COREQ; Tong et al., 2007).

### 2.3 Sample/participants

Participants were identified through wards specializing in the care of older people at two hospitals in the North of England. The first site provided medical care for older adult inpatients only and the second site combined orthogeriatric care (specifically older adults with neck of femur fragility fractures) and medical care for older adult inpatients. The sites were a convenience sample chosen to make best use of the time and resources available for data collection in the study.

#### 2.3.1 Study one (patient interviews)

Older people were approached about the study by a member of the research team (local research nurse or KH) during their hospital stay. Patients were eligible for inclusion in the study if they were; aged 65 years or older, had been admitted to one of the two older people wards with an unplanned admission of more than 5 days, able to speak English and, willing and able to be interviewed once at home by a researcher in 3 weeks of discharge. The 3 week window for interviews post‐discharge was chosen to facilitate accurate recall of hospital experiences. Patients were ineligible for inclusion in the study if they were unable to provide informed consent or they had been at home for more than 3 weeks at the point of interview. Where patients lacked the capacity to provide informed consent, the research team attempted to identify a relative, friend or carer who may wish to participate in the study. Relatives/friends/carers had to know the participant well and had to have visited them in hospital at least twice during their admission. We aimed to purposively sample patients to ensure balance in gender, age, ethnicity and level of frailty (as identified by Clinical Frailty Score; Rockwood et al., 2005).

#### 2.3.2 Study two (ward staff focus groups)

Ward staff members self‐identified an interest in participating in the study through responding to an invitation email/leaflet. Ward staff were eligible to participate in the study if they worked on or were linked to an older people ward (for patients aged 65 and over) for at least 3 months, and, were willing and able (with informal agreement from line manager to participate during work hours) to attend a 1‐h focus group. We aimed to purposively sample staff to participate in two focus groups at each hospital site. One of the focus groups aimed to include the perspectives of clinical healthcare workers (e.g. nurses, doctors, pharmacists, physiotherapists, occupational therapists, speech and language therapists, dietetic staff). The other focus group aimed to include the perspectives of non‐clinical staff (e.g. porters, domestic services staff, volunteers, administration staff).

### 2.4 Data collection

#### 2.4.1 Study one (patient interviews)

Semi‐structured interviews were conducted by one researcher (KH, male) inpatients/carers own homes between August and October 2019. Only the researcher and participants were present during interviews. Participants were not previously known to KH, except for where they had met during their hospital stay as part of recruitment for the study. KH is an experienced interviewer with a disciplinary background in Psychology (a Research Fellow educated to PhD level). Prior to the interview, participants were reminded of the aims of the study. No further information about the researcher's background was routinely provided. Topic guides were developed to capture risk factors which may have led to the development or exacerbation of MoF during hospital. The patient topic guide explored what a typical day was like during their stay on the ward; with prompts about getting dressed, bed rest, trips to the toilet, eating and medication. The carer topic guide explored relative's experiences of visiting the ward; with similar prompts about their observation of dressing, bed rest etc. The topic guides were not pilot tested.

#### 2.4.2 Study two (ward staff focus groups)

Focus groups were conducted in private rooms on the hospital wards between July and August 2019 by two researchers (KH and FW). Only the researchers and staff participants were present during focus groups. KH had previously been introduced to some ward staff as part of recruitment for study one and study two. FW (female) had not previously met ward staff prior to the focus groups. FW is an experienced qualitative and health services researcher with a disciplinary background in Psychology (a Research Fellow educated to PhD level). Staff were reminded of the aims of the study at the beginning of the focus group and KH/FW were introduced as health researchers. Topic guides were developed for the focus groups with clinical and non‐clinical staff (topic guides were not pilot tested). The topic guides for the focus groups with clinical staff asked about good practices in the ward for managing MoF and the challenges of managing each of the different MoF. The same topics were explored with non‐clinical staff, however, the terms for MoF were made accessible to a non‐clinical audience e.g. discussion of the challenges they had observed in caring for older people who are confused (delirium), have had a fall, struggling to move around or get out of bed (immobility) or people who may be struggling to eat or dress themselves (loss of function).

A copy of the topic guides is available in Supporting Information Files S1 and S2. Interviews and focus groups were audio recorded and transcribed verbatim. Fieldnotes were made to detail interruptions, distractions or additional thoughts on the topics discussed. Fieldnotes provided additional contextual data to inform the coding and interpretation of transcripts. Transcripts/findings were not returned to participants for comment and/or correction. At the end of data collection, the research team were satisfied that the interviews and focus groups had reached a point where little new information was emerging.

### 2.5 Ethical considerations

This study was approved by an NHS Research Ethics Committee in June 2019. All participants provided written informed consent. The right to withdraw from the study at any time without negative consequences was emphasized to all participants.

### 2.6 Data analysis

Data were analysed using directed content analysis; an approach which is useful for validating or extending existing knowledge or theory (Assarroudi et al., 2018; Hsieh & Shannon, 2005). From the scoping review of the literature we developed five broad categories of risk factors including;

1. Patient factors. Defined as any factor related to a characteristic of an individual patient e.g. age, illness severity, comorbidity, hearing impairment.
2. Environmental Factors. Defined as any factor related to a characteristic of the ward environment e.g. incorrect equipment, not having a clock or watch.
3. Process of Care Factors. Defined as specified processes that happen in the ward e.g. falls risk assessment, safety huddles.
4. Organizational Factors. Defined as factors which are influenced by the way in which care is planned or managed at a managerial or trust level e.g. staffing levels, duplicated assessments, bed availability.
5. Ward culture factors. Defined as customs or norms about the way in which the ward provides care e.g. leadership, multidisciplinary team (MDT) working, risk aversion.

Once familiarized with the data from each transcript, we looked at the data line‐by‐line to identify potential risk factors. Risk factors were coded inductively using terminology which stayed close to the original data and then organized as sub‐categories under one or more of the risk factor categories defined above. The coding framework was tested by two researchers (KH, FW) independently coding a sample of the data (1 focus group and 4 patient interviews). Discrepancies in coding were discussed and used to clarify the definitions for each category. Major discrepancies in coding were not noted. The remaining data were coded by one researcher (KH). Sub‐categories were reviewed and collapsed where there was overlap. Separate coding was completed for focus group and patient interview data and was facilitated by the use of NVIVO 12 software (QSR International Pty Ltd, 2018). The coded data were used to develop themes and a theoretical framework of modifiable risk factors.

#### 2.6.1 Theme development

Themes were developed to represent the richness and complexity of the qualitative data which underpinned the theoretical framework. To develop the themes, one researcher (FW) compared and contrasted the coded interview and focus group data in and across each of the main categories and between the staff focus groups and patient interviews. Theme development was based on the predominant risk factors identified by patients and staff and was an iterative process which included considerable back and forth between transcript data and theme organization. Simple mind maps were created to exemplify the relationships between the risk factors proposed in the theoretical framework. The wider research team discussed the themes to consider other perspectives on the data and clarify and refine interpretation of the data.

#### 2.6.2 Theoretical framework of modifiable risk factors

We developed a theoretical framework which draws on risk factors identified in the scoping review and from the qualitative study reported in this paper. The framework was developed logically to group risk factors together and also to specify proposed relationships between patient risk factors and related care management factors. Through discussions in the research team, risk factors were allocated to the predominant risk factor type (i.e. patient risk factors, linked care management risk factors and contextual risk factors) they were considered to belong to, though it is recognized that for some there maybe overlap between these. This was achieved by moving back and forth between the risk factors identified in the coded interview and focus group data and the theoretical framework and consideration of whether the risk factor was modifiable or not.

### 2.7 Rigour

Procedures to ensure rigour were incorporated throughout the study. These are described in Table 2 with reference to Lincoln and Guba's trustworthiness criteria (credibility, transferability, dependability and confirmability) which are commonly used to describe rigour in qualitative research (Guba, 1981; Lincoln & Guba, 1985; Schwandt et al., 2007).

**TABLE 2:** Procedures to promote rigour

Criteria	Definition	Description of steps taken in this study
Credibility	The extent to which an interpretation of data is representative of the experiences of participants	Discussion of data and themes with co‐author group (peer debriefing) to check that interpretations was representative of experiences. Co‐author group are from multidisciplinary backgrounds including: Psychology (KH, FW), Nursing (SC, DC), Medicine (ET), Physiotherapy (AF). Study findings were also presented to a public and patient involvement group consisting of five members (recruited from a local older people's action and support group). Participants suggested that findings relating to ward culture and staff shortages resonated with their own experiences. The group was also glad to see isolation and lack of stimulation was included as they felt strongly that this was a key factor in older peoples decline during a hospital stay.
Transferability	The extent to which findings might be applied or generalized to other participants in similar contexts	To inform readers judgements about transferability, we have included relevant contextual information about sites and participants in the findings.
Dependability	The extent to which a researcher's interpretation of data would be consistent if repeated	We used NVivo software to provide a clear audit trail for the analysis.
Confirmability	The extent to which the findings of the study are free from bias	Data were initially coded line‐by‐line using terminology which stayed close to the original data (and thus participant's experiences). In developing the themes, we actively explored atypical experiences to refine our interpretations. Two researchers coded a sample of transcripts (see method for further details) to ensure there was agreement on the coding of risk factors.

## 3 FINDINGS

A total of 26 patients and one carer provided consent to participate in an interview for the study. Nine patients dropped out of the study between providing consent and the interview being arranged. Reasons for dropout included; no longer wishing to participate, being unable to contact the patient or being unable to arrange the interview due to hospital readmission or the death of the patient. A total of 18 interviews were conducted with 17 patients and one carer (eight patients from site 1, nine patients and one carer at site 2). Table 3 shows an overview of patient characteristics. The mean age of patients was 79 (range 71–88) and the median CFS was 4 (range 2–7). All patient participants were of white ethnicity. The mean interview time was 38 min with a range of 17–67 min.

**TABLE 3:** Overview of participant characteristics

Participant number	Patient (P) or Carer (C) interviewed	Age	Sex	Clinical frailty score
01	P	72	Female	7
02	P	77	Male	6
03	P	71	Female	3
04	P	75	Female	3
05	P	77	Male	6
06	P	75	Female	3
07	C	86 (P)	Female (P)	7 (P)
08	P	78	Female	2
09	P	76	Female	4
010	P	88	Male	3
011	P	75	Male	2
012	P	85	Female	4
013	P	83	Female	5
014	P	73	Female	5
015	P	82	Female	2
016	P	85	Male	5
017	P	81	Female	3
018	P	79	Female	7

A total of five focus groups were held (three at site 1 and two at site 2). Seven clinical staff participated at site 1 (two doctors, a staff nurse, a pharmacist, an occupational therapist, physiotherapist and health care assistant) and five clinical staff at site 2 (An occupational therapist, a trainee nurse, a doctor, a health care assistant and a technical instructor). Five non‐clinical staff participated at site 1 (three domestic staff, one tea server, one volunteer) and five non‐clinical staff at site 2 (two volunteers, one ward clerk, two porters). A further three staff at site 1 (two clinical, one non‐clinical) and five staff at site 2 (three clinical, two non‐clinical) provided informed consent but were unable to attend the focus group at the arranged time. The mean length of time for the focus groups was 47 min with a range of 28–57 min.

**FIGURE 1:**
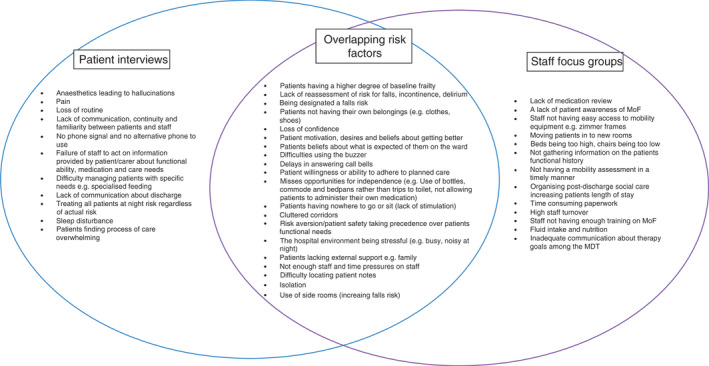
Risk factors identified in the patient interviews and staff focus groups

In total, we identified 44 risk factors from the coding of interview and focus group data; including 11 risk factors from the patient interviews, 13 risk factors from the focus groups with staff and 20 risk factors identified in both patient interviews and staff focus groups. Figure 1 shows the risk factors identified in the patient interviews and staff focus groups and the overlapping risk factors reported by both patients and staff.

### 3.1 Themes

Three themes were developed from the interview and focus group data.

1. Promoting independence and balancing risk

Promoting patient independence in the ward by encouraging patients to move around and do things for themselves, wherever possible, was identified by clinical staff as a key mechanism for reducing the development or exacerbation of MoF. However, clinical staff reported that promotion of independence needed to be balanced with some assessment of risk as encouragement of independence in some areas for example, general mobility, independent toileting could increase levels of risk in others for example, falls. Some clinical staff expressed fear of organizational repercussions for falls and this led to staff erring on the side of caution when it came to promoting independence.

Everywhere I have worked falls has been a massive drive to reduce falls…even if someone maybe just been spotted being a little bit unsteady at one point… it’s just kind of like, okay, don’t get up on your own, press your buzzer and I think it’s that kind of thing and again, it’s just because of that fear… (clinical focus group, site 1)

Patients also alluded to this issue when they indicated they felt the need to seek permission to move independently or go to the toilet by themselves. Some indicated they did not get out of bed during their hospital stay and had to conform with what others were doing on the ward:

… you just slept all day … we weren't encouraged to do anything and you fall into that trap, you're trapped. It's almost like being imprisoned in a way. You're there and you just conform … (patient interview, p. 15)

Clinical and non‐clinical staff discussed whether there was clear communication with all patients throughout their stay about moving around or to encourage independence:

I think they may feel that they’re not allowed to do this, and it’s, you know, the olden days where you stayed in bed… (non‐clinical focus group, site 1)

we probably maybe better at promoting what not to do rather than what you can do. (clinical focus group, site 1)

However, there were examples where patients reported being more independent in terms of getting dressed or moving around the ward. Facilitators to this included encouragement and clear communication from ward staff and the patient's own ability to ambulate without needing assistance, level of motivation and willingness to push the boundaries.

So I was using a commode, they were bringing a commode and wheeling me, and then they started to wheel me across to the toilet. And then I thought, where does this go now? I'm sure I'll be able to walk across to the toilet, you know, meself. So I, I just said, "Can I chance it?", "Well, I thought you had a commode", I said, "Yes, I have been, but I just wondered if I can chance going meself, with a bit of help". (patient interview, p. 18)

A barrier to promoting independence reported in the focus groups was staffing levels. For example, not having time to support patients to walk to the bathroom (so using a commode instead).

…like if we’ve seen a patient and said they can walk ten metres, so they could walk to the bathroom, the healthcares and the nurses are so busy on the ward that actually it’s easier to just transfer them onto a commode, wheel them to the bathroom because it saves time, so it’s not giving them the opportunity to mobilise, but because of staffing… (clinical focus group, site 2)

A further barrier to promoting independence was a lack of equipment e.g. walking frames to help patients to mobilize. Delays to accessing such equipment were sometimes also due to delays in accessing an assessment from a physiotherapist. However, some staff hinted at a misconception that a physiotherapist assessment was needed;

P1: I think then it's that reliance on they need a physiotherapist.

P2: Mm, but then we can, rehab support workers can assess for walking aids but none of them do, do they?’ (clinical focus group, site 1)

Clinical staff also reported missed opportunities to promote independence when the assessment of risk level was not updated in a timely manner.

Yeah, it almost needs to be like reassessed and reassessed. Like they’ll put a falls alarm on patients for like post‐op delirium but when that… I mean, sometimes they don’t even have the delirium or when it resolves it’s not, right, let’s take this off now, you know, and then the patient, that’s driven into them, I can’t get up on my own, you know… (clinical focus group, site 1)

2. Personalized communication

Communication was discussed by both patients and clinical staff. For clinical staff, good communication was a key element of recognizing and ameliorating the risks for frailty and was facilitated by multidisciplinary team working, safety huddles, nursing rounds and collecting in depth medical and functional history.

Every senior review, every ward round, mobility, falls, stuff like that, are included within seeing the patient…have they been out of bed, are they eating okay, you know, what are their bowels like, you know, those are checked daily…it’s a holistic approach, and not just from when they come in but it’s a continual holistic approach, just watching their progress as they go. (clinical focus group, site 1)

However, this sense of communication, knowledge and personalization of care was not always apparent to patients. Some described feeling ‘anonymous’ (patient interview, participant no. 10) or felt as though some staff were not aware of what they could or could not do;

…but the care assistants…don't know anything about that person in the bed. And I think they should do, I don't mean they should know everything, but I think they should know what that patient's capable of doing, you know, can they eat by themselves or, but they don't seem to know that….I really did pick up on that, I thought, you don't know anything about me’. (patient interview, participant no. 18)

Those with specific care requirements (e.g. those requiring assistance with feeding) were described by patients as being at particular risk of not having their needs met due to the lack of continuity or information sharing between staff. For example, one carer described advising ward staff how best to feed their client who had dementia but felt that this information was not shared between staff:

You know, the times that I’ve said to them, if you did this and if you did it that way, she’d be alright. And they said, what do you mean, so I’d show them and it were like, for feeding her, it were just, you just touch the lip here and say ‘[name of patient] open’ and then she’d open her mouth and take the food and they’d go [gasps]. So, and the number of nurses that I told, you know, if I met different ones, still the same. (carer interview, participant no. 7)

The relational elements of communication were particularly important to patients; for example, having a sense of familiarity with the staff was important for facilitating information exchange. A barrier to communication was being in a side room as this limited opportunities for patients to get to know staff and ask questions:

I told them, I just feel like I’ve been chucked in a field here, I said, because nobody's coming and telling me anything, nobody's, I don't see anybody, you couldn’t see anybody and you couldn’t see out or anything like that… (patient interview, participant no. 6)

Clear and personalized communication could act as a facilitator to reduce risk for some MoF. For example, a patient shared how they were encouraged to walk up and down the ward by themselves:

…then eventually they came and they said, you know, 'you can walk about, get up and walk about by yourself then', so I was able to walk around my little ward with the Zimmer frame and keep walking and practising walking and sitting down and resting. (patient interview, participant no. 4)

Conversely, some patients awaited instructions from ward staff when it came to what they should and should not be doing in terms of moving around or being independent. However, this communication was sometimes ad hoc:

I struggled to get out of bed and one of the nurse saw me, “what are you doing? Get back into bed, you’re not allowed to walk, you want anything there’s a buzzer here, buzz for a nurse and a nurse will come”, so [figurative] slapped wrist. (patient interview, participant no. 15)

3. Loss of routine, stimulation and confidence

Patients described how their admission to hospital was a complete change to their normal routine, with an immediate loss of independence when it came to activities of daily living such as dressing, moving around or preparing meals.

If you were at home you get up, you get washed, you get dressed, you come down, you have your breakfast and that routine, that normality, goes because you're just in the bed. (patient interview, participant no. 11)

This was described by a carer and by staff as being problematic particularly for those with some existing level of frailty; with loss of independence and routine increasing the risk of losing function in activities of daily living. Patients and staff also reported that there was very little in the way of other activity for patients on the ward. Some patients expressed a sense of monotony, boredom and also isolation. For example, the quotation below is from a patient who described feelings of isolation after they were unable to speak to their family:

And I’d taken my mobile phone, but it wouldn't work… they were very busy, because that was the admissions unit and the nurse there did say “oh, don’t worry, they will have informed your daughter you know, where you are”. So, I knew that she would have been told, but it's not the same as being able to ring people…you lie there thinking, oh she’ll be so worried… So, you know, after two or three days I did see people, but I did feel it was very isolating and that's not good. That’s not good. (patient interview, participant no. 18)

Non‐clinical staff, in particular, raised the issue of loneliness and lack of stimulation for some patients who did not have visitors or who were in side rooms. One volunteer recognized this to be a risk for delirium. The volunteer was able to spend time talking to patients in their role but suggested that low staff levels meant that nursing staff may be unable to do the same:

Well on an elderly ward you need more people who are like me…somebody sitting talking to somebody prevents delirium…and a nurse can't spend two hours preventing delirium but that's what's needed. (non‐clinical focus group, site 1)

For some participants having a period of dependency whilst in hospital led also led to a loss of confidence.

…when I was leaving I thought 'ah, how am I going to cope without them' and this is me who just did whatever I wanted, I did the same now as I did when I was twenty, you know, I just did anything I wanted, and, and [now] I'm thinking 'how am I going to cope without them'… (patient interview, participant no. 4)

Loss of confidence was identified in focus groups and interviews as a factor which may lead to the development or exacerbation of MoF in the hospital setting. Clinical and non‐clinical staff also related loss of confidence to immobility; with patients moving around less due to fear of falling. The quotation from a member of non‐clinical staff below also suggests that patients may feel broader uncertainties about whether moving around is the right thing for their recovery:

Yes, it is, a lot of it is from fear, because they're frightened of falling, they're frightened of, you know, doing the wrong thing… (non‐clinical focus group, site 1)

However, one member of clinical staff also wondered whether their own processes for safely mobilizing patients may also exacerbate loss of confidence:

Or like even if we’re like mobilising patients and we’re clinging on to them like that, it does nothing for anyone's confidence, you know, them thinking I need someone to be on my hip the whole time, rather than if you just take a step back and you know. (clinical focus group, site 2)

### 3.2 Theoretical framework of modifiable risk factors for the development or exacerbation of MoF in the hospital setting

**FIGURE 2:**
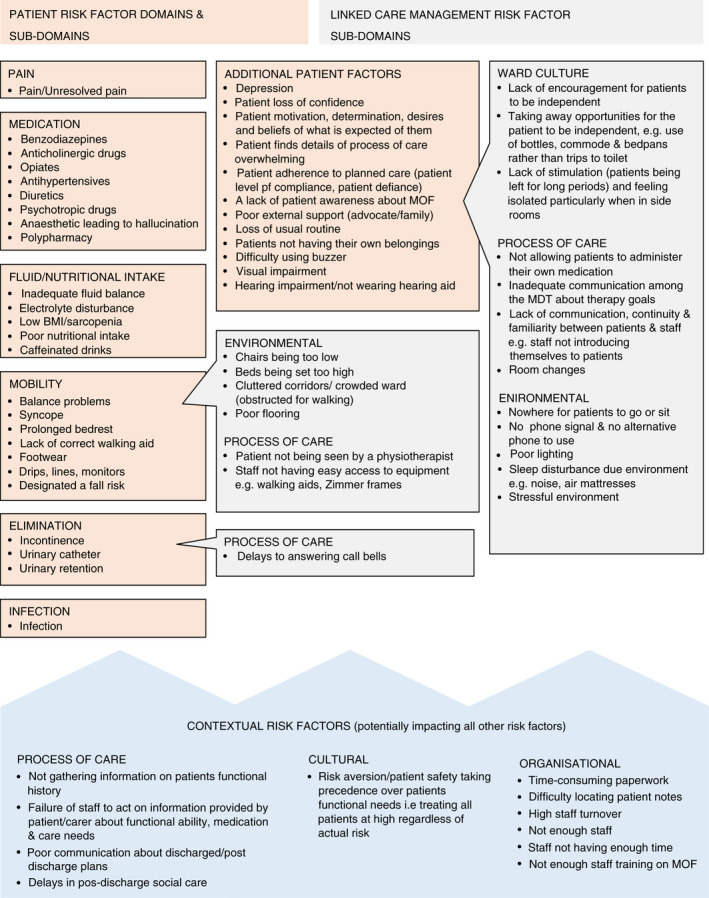
Theoretical framework of risk factors for the development or exacerbation of MoF in the hospital setting

Figure 2 shows the theoretical framework we developed based on the modifiable risk factors identified in this qualitative study and the previous scoping review.

There was some overlap between the risk factors identified in the scoping review and the qualitative work, for example, pain, sleep disturbance. In addition to the risk factors identified in the scoping review, this qualitative study contributed a further 36 risk factors to the theoretical framework. Table 4 shows the additional risk factors identified by this qualitative study included in the framework. Table 4 shows that many of the risk factors identified by the qualitative study were related to linked care management risk factors such as ward culture, process of care and organizational factors.

**TABLE 4:** Additional risk factors identified by this qualitative study

Domain and sub‐domain in theoretical framework	Risk factor
Medication	Anaesthetic leading to hallucination
Mobility	Being designated a falls risk
Mobility (environmental)	Chairs being too low
	Beds being set too high
	Cluttered corridors/crowded ward obstructed for walking
Mobility (process of care)	Patient not being seen by physiotherapist
	Staff not having easy access to equipment e.g. walking aids, Zimmer frames
Additional patient factors	Patient loss of confidence
	Patient motivation, desires and beliefs of what is expected of them
	Patient finds details of process of care overwhelming
	Patient adherence to planned care (patient level of compliance, defiance)
	A lack of patient awareness about MoF
	Poor external support (advocate/family)
	Loss of usual routine
	Patients not having their own belongings
	Difficulty using buzzer
Additional patient factors (ward culture)	Encouraging patients to be independent is not the norm or encouraged by leadership
	Taking away opportunities for the patient to be independent (e.g. use of bottles, commodes and bedpans rather than trips to the toilet)
	Lack of stimulation (patients being left for long periods) and feeling isolated particularly when in side rooms
Additional patient factors (process of care)	Not allowing patients to administer their own medication
	Inadequate communication among the MDT about therapy goals
	Lack of communication, continuity and familiarity between patients and staff (e.g. staff not introducing themselves to patients)
Additional patient factors (environmental)	Nowhere for patients to go or sit
	No phone signal and no alternative phone to use
	Stressful environment
Contextual risk factors (process of care)	Not gathering information about the patients functional history
	Failure of staff to act on information provided by patient/carer about functional ability, medication and care needs
	Post discharge care and communication lacking
	Delays in post discharge social care
Contextual risk factors (cultural)	Risk aversion/patient safety taking precedence over patient's needs that is, treating all patients as high risk regardless of actual risk
Contextual risk factors (organizational)	Time consuming paperwork
	Difficulty locating patient notes
	High staff turnover
	Not enough staff
	Staff not having enough time
	Staff not having enough training on MoF

In the theoretical framework we have categorized the risk factors into patient risk factor domains (e.g. mobility) and sub‐domains (e.g. balance) and linked care management risk factor sub‐domains, (e.g. poor flooring). The linked care management sub‐domains were associated with the environment, ward culture and processes of care. We also theorized that some contextual risk factors had the potential to impact patient and linked care management risk factors for example, not having enough staff may lead to delays in answering call bells, being risk averse may hamper encouraging patients to be independent.

## 4 DISCUSSION

We have developed a theoretical framework consisting of 67 modifiable factors which may contribute to the development or exacerbation of MoF in the hospital setting (and subsequent physical and functional decline). A total of 36 additional modifiable risk factors (which were not previously identified in our scoping review of the literature) were identified through the qualitative interviews with patients and focus groups with ward staff. Many of the modifiable risk factors identified were related to the context of care management such as the ward culture, process of care and organizational factors. The theoretical framework developed maps out the potential associations between patient risk factors (e.g. loss of confidence), and linked care management risk factors which may contribute to increased levels of patient risk (e.g. lack of encouragement to be independent).

Although iatrogenic risk factors relating to the process or organization of hospital care have been recognized as important, they are less well defined in comparison to risk factors related to the characteristics of individual patients (Lafont et al., 2011; Sourdet et al., 2015). Recommendations for risk assessment often consider risk at an individual rather than organizational level (National institute for Health & Care Excellence, 2019a, 2019b, 2019c); for example, by taking into consideration a patient's age or cognitive function rather than reducing delays to answering call bells or addressing the lack of access to equipment. The findings of this study suggest the importance of addressing risk factors at both an individual and organizational level to reduce the risk of functional decline in hospitalized older adults.

In addition, the findings suggest that it is important for organizations to consider risk as a whole, across different MoF. For example, staff in this study reported that organizational drivers to reduce falls led to them erring on the side of caution when it came to encouraging patients to move around the ward independently. However, the resulting restriction of movement may lead to increased risks for other MoF for example, immobility, delirium; highlighting the potentially adverse consequences of organizational safety initiatives to reduce falls (Growdon et al., 2017). Growdon et al. (2017) challenge the idea that increased mobilization necessitates an increased risk of falls. For example, the Hospital Elder Life Programme (HELP; aimed at reducing levels of delirium), encourages mobilization and a recent systematic review and meta‐analysis suggested that this programme may also reduce the number of falls (Hshieh et al., 2018). Organizations should take a balanced approach when encouraging falls prevention, given the risk for such initiatives (including requirements for falls reporting and the use of falls as a marker of care quality) to cause longer‐term harm by restricting movement in hospitalized older adults (Growdon et al., 2017). A co‐ordinated approach to managing MoF is needed to ensure that top‐down initiatives or audits do not create organizational barriers to reducing risk.

A further challenge to reducing risk factors for MoF is the impact of low staffing levels which were reported to be problematic by both patients and staff. In this busy and understaffed context, there were missed opportunities to promote independence on the ward for example, the use of commodes rather than supporting patients to walk to the toilet. This finding suggests that low staffing levels may further contribute to the restriction of movement (and thus the development or exacerbation of MoF) in hospital wards. Low staffing levels were also reported to contribute to lower quality communication between patients and staff; a finding which has been echoed in other qualitative studies (Bridges et al., 2020). Care processes (e.g. MDT meetings, safety huddles) are often geared towards facilitating information exchange between staff but there were less clear processes to ensure information exchange and relationship building between ward staff and patients (and their families). Such exchanges may be vital for staff to promote independence and help patients (and their families) to understand what they can do to decrease their level of risk for MoF (D’Avanzo et al., 2017; Gray et al., 2016).

Some interventions have overcome staffing barriers by upskilling other members of the health care team, involving family members or using volunteers to reduce the risks for MoF (Fox et al., 2012; Hshieh et al., 2018). However, given the variation in processes of care across hospital systems (and associated organizational risk factors), it may also be necessary to develop fully contextualized interventions which take into account local barriers and assets to facilitate risk reduction (Liu et al., 2013; Moore et al., 2014). For example, some hospitals may have access to a volunteer workforce which could be used, others may use networks of staff champions to facilitate change. Such an approach may include the generation of toolkits for services to assess their current provision and target change based on this (Conroy et al., 2019).

By considering risk factors for MoF as a whole, it may also be possible to reduce overlapping care processes (e.g. multiple risk assessments) to maximize staff time and minimize inefficiencies caused by multiple care pathways for different MoF (e.g. duplicative paperwork). Redley and Raggatt (2017) found that standardized risk assessment forms for older people were often duplicated by different members of the MDT. Staff also reported a high level of administrative burden associated with completing such paperwork (Redley & Raggatt, 2017). To reduce duplicative processes, it is important to clearly define the role of different MDT members in identifying and reducing risk for MoF. Nurses already play a significant role in risk screening and assessment procedures (Han et al., 2021; Redley & Raggatt, 2017), care quality and patient safety (Aiken et al., 2017) and therefore may be well placed to co‐ordinate risk reduction for MoF. However, a need for further specialist or advanced nursing training to support leadership and competency in working with older people with frailty has been identified (Goldberg et al., 2016; Naughton et al., 2016). Further specialist training and competency frameworks may also be required for other healthcare professionals to facilitate risk reduction (Roller‐Wirnsberger et al., 2020; Windhaber et al., 2018).

The theoretical framework developed as part of this study provides an overarching model of modifiable risk factors which may reduce the development or exacerbation of MoF in hospitalized older adults. The framework will aid the development of a comprehensive and targeted system of care designed to reduce overlapping care processes and address the risk factors identified. The theoretical framework indicates the importance of organizational and contextual factors suggesting that it may be useful to draw on existing theories for example. The Consolidated Framework for Implementation Research, Normalization Process Theory (Damschroder et al., 2009; Murray et al., 2010) to support the implementation of the intervention in complex healthcare systems. Work to prioritize the modifiable risk factors to be targeted as part of the system of care have been undertaken and is reported in separate paper.

### 4.1 Limitations

Due to the limited time available for recruitment, we did not fully achieve our purposive sampling strategy. For example, the sample did not include patients from different ethnic groups and there were more female than male participants. The views and experiences of patients from these groups may be different from those who participated in this study. In addition, although we recruited from two NHS services, the services were in one geographical location and thus, the experiences of participants may not represent other areas of the country or countries outside of the United Kingdom. We did not note major differences between the sites in terms of patient or staff experiences or the risk factors identified, however, this was not explored formally in the analysis. The analysis was also limited in specifically exploring other nuances of the data including the impact of reason for admission on patient experience. Lastly, it is important to note that this work was undertaken prior to the COVID‐19 pandemic; which is likely to have had a significant impact on the way in which care for hospitalized older adults is organized and delivered.

## 5 CONCLUSION

To reduce the risk of functional decline in hospitalized older people, it is necessary to have a comprehensive understanding of modifiable risk factors which may contribute to the development of exacerbation of MoF. It is also important to recognize the complexity of the healthcare systems in which risk is managed and to understand the ways in which the process or organization of hospital care contributes to increasing or mitigating risk for MoF. The theoretical framework developed in this study will act as a starting point for developing a novel system of care to reduce the risk of loss of independence in hospitalized older adults. The framework will be subject to further validation and development as part of this work. A future programme of research will be undertaken to refine and evaluate the effectiveness of the developed system of care.

## CONFLICT OF INTEREST

No conflict of interest has been declared by the authors.

## AUTHOR CONTRIBUTIONS

This study was conceived of and designed by Elizabeth Teale, David Clarke, Susanne Coleman and Kristian Hudson. Data collection was undertaken by Kristian Hudson and Faye Wray. The coding and analysis of qualitative data were primarily undertaken by Kristian Hudson and Faye Wray. All authors contributed to the interpretation of data. The manuscript was drafted by Faye Wray and revised in line with comments and contributions from Susanne Coleman, David Clarke, Anne Forster, Kristian Hudson and Elizabeth Teale. All authors approved submission of the manuscript.

### PEER REVIEW

The peer review history for this article is available at https://publons.com/publon/10.1111/jan.15120.

## Supporting information

Supplementary Material

Supplementary Material

## Data Availability

The data that support the findings of this study are available on request from the corresponding author. The data are not publicly available due to privacy or ethical restrictions.
