# Supplementary material for: Risk factors for manifestations of frailty in hospitalized older adults: A qualitative study
Source: J Adv Nurs. 2021 Nov 30;78(6):1688–703. doi: 10.1111/jan.15120 (PMC9299686; doi:10.1111/jan.15120)
Supplement: Supplementary file 1 — Supplementary Material [file JAN-78-1688-s001.doc]

**Supporting information file 1:** Topic guides for patient and carer interviews

**_______________________________________________________________________________________________**

**Topic guide for patient interviews**

Introduction for patient interviews

Thank you for agreeing to take part in this interview. I would like to talk to you today about **your recent experiences** whilst in (hospital name/ward) and how these might have helped you to be independent and **do things for yourself**, or might sometimes have made it more difficult for you to be independent and do things for yourself. We are also interested in your views and which aspects of your care in hospital were most important to you. We would like to know what you think the hospital staff could do to help people like yourself to be independent and do things for themselves as far as they are able.

If at any point you would like to stop or take a break, just let me know. Don’t worry if you feel like you can’t think of anything to say, there are no right or wrong answers. I may prompt you to talk about some things in a little more detail, but don’t worry if you feel you can’t or do not wish to answer any further.

**Are you happy for me to audio record this interview?**

Although I will be audio recording this interview everything you say is confidential, and nothing will be linked back to you. You can also let me know if you wish to stop the interview at any time.

**Do you have any questions before we start?**

**Is it OK to start the interview now?**

| **Questions** | **Prompts** | **MoF** |
| --- | --- | --- |
| 1) Can you tell me about what led to you spending some time in hospital name/ward number? | Length of hospital stay / impact of illness / extent of event i.e. full recovery or ongoing impairments or problems? |  |
| 2) One thing that might be useful is to go through a typical day… so you were admitted to the ward because/ for (reason)… | Was that the first thing that happened? When did you get up? Did you need help? What kind of help did you need and were the staff able to provide that?  Prompt for person centred care e.g. Did you have a choice in how you washed yourself? Did staff let you wash in a way you wanted or did you have to do it differently?  Keep checking how independence during the recent hospitalisation compares with prior to hospitalisation. “So before you were in hospital you could wash yourself, but now you can’t? | Imm  LoF |
| 3) How did you manage with things like getting dressed? Did you need any help | How much did the staff help you? How much did you need the staff? If mobility doesn’t come up ask afterwards… Were you able to get out of bed (around the bay/side room) yourself? If needed, what kind of help did you need?  Prompt for units philosophy of care e.g. If they were told to wash or do something a certain way ask them “did staff explain why?” | LoF |
| 4) How did you manage with your meals? | What did the nurses do at mealtimes? If you needed help, how available were staff?  If a participant cannot remember which staff they saw, they may remember what they were wearing which will help identify who they mean. | LoF |
| 5) How did you manage with your mobility? | Did you get the help you need? If you wanted to walk somewhere were you allowed to? Did you have contact with physiotherapists or OT’s? What kind of uniform were staff wearing? | LoF  Imm |
| 6) Were you or the staff concerned about falls? Did you have any falls? | If this was a concern were any restrictions advised or required by the staff | LoF  Fal |
| 7) Were you able to manage getting to and from the toilet etc Did you have any problems using the toilet? | Check mobility. How much did the staff help you? How much did you need the staff? Were you encouraged to use the toilet say every two hours for example or were you prompted to tell staff when you had used? Did you make the decisions, be involved in decisions, or did the staff?  Keep checking how independence during the recent hospitalisation compares with prior to hospitalisation. | LoF  Inc  Imm |
| 8) You told me about washing, dressing, meals, mobility, falls.. Did you need other kinds of help from the nursing staff or other staff? | Slippers, clothing, seeing relatives, answering questions, having time for you.. | Any |
| 9) Experience of pain, impacts of a medical condition: Were you limited/have problems with pain, breathing, infection etc. How did this impact your experience? | What kind of help did you need and were the staff able to provide that? | Any |
| 10) Contrasting night and day: How did you manage during the night? | What kind of help did you need and were the staff able to provide that? | Del  Fal |
| 11) Dignity: Overall, how were you treated by the staff? Did you feel respected? | Prompts: Were there any things that concerned you related to personal privacy or maintaining your dignity? |  |
| 12) Emotional and Psychological support: Did you feel supported emotionally? E.g. If you were feeling sad, angry, upset did you feel like someone was there to help? | Were staff aware of how you were feeling? | Del |
| 13) So just looking back at your overall experience what were the things you disliked? | Was there anything which was really unhelpful? (Become more general at the end). This might overlap with Q:12 so just be mindful no need to repeat if addressed at 12. |  |
| 14) What things did you like? | In terms of helping older people in the ward, perhaps someone with the same condition as you, how helpful were the staff? Helpful? Unhelpful? What did the staff do well in your opinion? |  |

Fal – Falls Imm - Immobilty

Del – Delerium Inc - Incontinence

LoF – Loss of function

**Topic guide for carer interviews**

Introduction for carer interviews

Thank you for agreeing to take part in this interview. I would like to talk to you today about **your friend/relatives** **recent experiences** whilst in (hospital name/ward) and how these might have helped them to be independent and **do things on their own**, or might sometimes have made it more difficult for them to be independent and do things without help. We are also interested in your views and which aspects (name) stay in hospital were most important to them. We would like to know what you think the hospital staff could do to help people like (name) to be independent and do things for themselves as far as they are able.

If at any point you would like to stop or take a break, just let me know. Don’t worry if you feel like you can’t think of anything to say, there are no right or wrong answers. I may prompt you to talk about some things in a little more detail, but don’t worry if you feel you can’t or do not wish to answer any further.

**Are you happy for me to audio record this interview?**

Although I will be audio recording this interview everything you say is confidential, and nothing will be linked back to you. You can also let me know if you wish to stop the interview at any time.

**Do you have any questions before we start?**

**Is it OK to start the interview now?**

| **Questions** | **Prompts** | **MoF** |
| --- | --- | --- |
| 1) Can you tell me about what led to (name) spending some time in hospital name/ward number? | Length of hospital stay / impact of illness / extent of event i.e. full recovery or on-going impairments or problems? |  |
| 2) One thing that might be useful is to go through a typical day for (name).. Were you able to visit them often? | Check when visiting times are for the ward and how often the carer attended.  What did (name) tell you about getting up in the morning? Were they able to get up? Did they need help? Did they wash themselves? What kind of help did they need and were the staff able to provide that?  Did you notice any difference once they got in? Any difference in how they washed?  Prompt for person centred care – Did (name) have a choice in how they washed? Did they have to do it differently?  Keep checking how independence during the recent hospitalisation compares with prior to hospitalisation. “So before they were in hospital they could wash independently, but now they can’t? So last week before their fall they were able to.. but now they..” | Imm  LoF |
| 3) How did (name) manage getting dressed? | Did they say how much the staff helped? How much did they need the staff? If mobility doesn’t come up ask afterwards.. Were they able to get out of bed (around the bay/side room) on their own? If needed, what kind of help did they need?  Prompt for units philosophy of care.  If staff told them to get stay in bed and not get dressed did (name) say they explained why? | LoF |
| 4) How did they manage with their meals? | What did they say the nurses did at mealtimes? If they needed help how available were staff? Did their eating habits change once they were admitted? | LoF |
| 5) Did they have any problems with their mobility? | Did (name) get the help they needed? If they wanted to walk somewhere were you allowed to? Did you see them having contact with physiotherapists or OT’s?  If a participant cannot remember which staff they saw, they may remember what they were wearing which will help identify who they mean | LoF  Imm |
| 6) Were you or the staff concerned about falls? Did (name) have any falls? | If this was a concern were any restrictions advised or required by the staff? | LoF  Fal |
| 7) Was (name) able to manage getting to and from the toilet etc Did they have any problems using the toilet? | Check mobility. How much did the staff help them? How much did they need the staff? Did they tell (name) how toilet trips would work or did (name) have some say? E.g. were they encouraged to use the toilet say every two hours for example or were they prompted to tell staff when they had used it? Did they make the decisions, be involved in decisions, or did the staff?  Keep checking how independence during the recent hospitalisation compares with prior to hospitalisation. | LoF  Inc  Imm |
| 8) You’ve told me about (name’s) washing, dressing, meals, mobility, falls..Did (name) need any other type of help from the nursing staff or hospital staff? | Slippers, clothing, seeing relatives, answering questions, staff having time for (name).. | Any |
| 9) Experience of pain, impacts of a medical condition: Was (name) limited/have problems with pain, breathing, infection etc. How did this impact their experience? | What kind of help did they need and were the staff able to provide that? | Any |
| 10) Contrasting night and day: Do you know how (name) managed during the night? | What kind of help did they need and were the staff able to provide that? | Del  Fal |
| 11) Dignity: Overall, how do you think (name) was treated by the staff? Did you feel they were respected? Did you talk to them about this? | Were there any things that concerned you or (name) relating to personal privacy or (name) maintaining their dignity? |  |
| 12) Emotional and Psychological support: Did (name) feel supported emotionally? E.g. If (name) feeling sad, angry, upset did they feel like someone would have been there to help? | Were staff aware of how (name) was feeling? Did their mood stay the same or worsen over time? Did you feel you needed to be there? | Del |
| 13) So just looking back at (name’s) overall experience what were the things they/you disliked? | Was there anything which was really unhelpful? (Become more general at the end). This might overlap with Q:12 so just be mindful no need to repeat if addressed at 12. |  |
| 14) What things did they/you like? | In terms of helping someone with the same condition as (name), how helpful were the staff? Helpful? Unhelpful? What did the staff do well in your opinion? |  |

Fal – Falls Imm - Immobilty

Del – Delerium Inc - Incontinence

LoF – Loss of function
