# Supplementary material for: Risk factors for manifestations of frailty in hospitalized older adults: A qualitative study
Source: J Adv Nurs. 2021 Nov 30;78(6):1688–703. doi: 10.1111/jan.15120 (PMC9299686; doi:10.1111/jan.15120)
Supplement: Supplementary file 2 — Supplementary Material [file JAN-78-1688-s002.docx]

**Supporting information file 2:** Topic guide for Clinical and Non-Clinical staff focus groups

**__________________________________________________________________________________**

**Focus group topic guide – Clinical Staff**

**Introduction and set-up for focus group**

1. Brief introductions (amongst participants and researcher)
2. Thank you for taking the time out to be here. We really appreciate it.
3. We are interested in **your experiences** of treating/ caring for/ supporting frail older adults during episodes of inpatient care.
4. Our interest is around **Manifestations of Frailty**. What we mean by manifestations of frailty is **falls, delirium, immobility, incontinence and loss of function** and how these can develop in older patients while they stay on a hospital ward.
5. We know what some of the causes of frailty are but there’s a lot we don’t know and we’re interested in your experiences because you’ll know things we as researchers just don’t know.
6. We’re not here to test you, this is about what you’ve seen and experienced. There’s no right or wrong answers. Our job here is not to judge, but rather put you centre stage.
7. We think this will take about an hour, we don’t think it will run over and will keep an eye on the time.
8. Has anyone got any questions before we start?

| **Clinical Staff Focus Group Questions** |  |  |
| --- | --- | --- |
| **Questions** | **Prompts** | |
| 1. What do you think ward X does well in terms of promoting and maintaining patients independence? | What initiatives do you have in place to help maintain and promote the independence of patients while on the ward? E.g. medication, toilet trips, moving around the ward, self-care, meal-times.  Prompt for both job specific influences/responsibilities but also wider more general initiatives e.g. ending PJ paralysis.  Huddles?  Group feedback sessions?  What do they do to assess? What do they do individually? What do they do collectively? What strategies have they devised to address the issues? | |
| 2. Are there any things that don’t go so well? | Anything that may decrease independence? E.g. medication, toilet trips, moving around the ward, self-care, meal-times.  Staffing?  Equipment?  Patients with specific care needs e.g. dementia, low mobility. | |
| 3. So what are some of the specific challenges around managing people with delirium, falls risk, incontinence, immobility, loss of function? | What’s the most challenging thing?  What’s the most difficult thing to prevent?  Summarise what they have mentioned in Q1 and 2 to draw more out if necessary | |
| 4. Given that we’ve recounted some of the challenges around maintaining and promoting the independence of patients on the ward, if you could change one thing to make things better what would it be? |  | |

**Focus group topic guide- Non-Clinical staff**

**Introduction and set-up for focus group**

1. Brief introductions (amongst participants and researcher)
2. Thank you for taking the time out to be here. We really appreciate it.
3. We’re keen to talk to you about your experiences of working with frail older adults during their time in hospital.
4. We know that older patients can become increasingly frail while staying on a hospital ward. There are some problems we know are common. These are inpatients getting confused (delirium), having falls (falls), not moving very much, for example staying in bed all day (immobility), problems using the toilet on their own (incontinence) and people ending up being able to do less day to day activities, than before they came into hospital for example getting dressed (loss of function). The poster shows these.
5. Our interest is in understanding the challenges the ward faces in helping people maintain their independence while in hospital, and hearing about anything you have noticed which might help promote patients’ independence. There are things you’ll know that we as researchers simply don’t because you spend time here.
6. We’re not here to test you, we want to hear about what you’ve seen and experienced. You help things happen, see things, perhaps that other ward staff don’t see. There’s no right or wrong answers we just want to know about your experiences.
7. Has anyone got any questions before we start?

**Non-Clinical Staff Focus Group Questions**

| **Questions** | **Prompts** |
| --- | --- |
| 1. What do you think ward X does well in terms of promoting and maintaining patients independence? | Focus on maintaining patients independence generally.  What stands out for them? What things do they notice routinely that could be improved? What have they seen? Increases/decreases in independence? E.g. medication, toilet trips, moving around the ward, self-care, meal-times. |
| 2. Are there any things that don’t go so well? | Is there anything the unit does which seems to decrease patients’ independence? E.g. ward routines, staffing levels, patients having access to the things they need. |
| 3. What are some of the specific challenges this unit seems to face around caring for people who are confused (delirium), have had a fall (falls), have problems with going to the toilet (incontinence), struggling to move around or get out of bed (immobility), or who may be having difficulty dressing themselves, eating (loss of function)? | What’s the most challenging thing?  What could be done to change it?  Summarise what they have mentioned in Q1 and 2 to draw more out if necessary. |
| 4. Given that we’ve recounted some of the challenges around maintaining and promoting the independence of patients on the ward in the face of five main problems we’ve discussed i.e. falls, delirium,.. if the ward could change one thing to improve how it works with older people with frailty what do you think it should be? |  |
